# Polysaccharide Hydrogels

**DOI:** 10.3390/gels5030038

**Published:** 2019-07-29

**Authors:** Bjørn Torger Stokke

**Affiliations:** Department of Physics, NTNU—Norwegian University of Science and Technology, NO-7491 Trondheim, Norway; bjorn.stokke@ntnu.no

Polysaccharides are a unique source of organic materials in terms of abundance, structural diversity and functionalities. Alongside nucleic acids and proteins, the collective group of polysaccharides makes up the macromolecules serving as the fundamental components of living organisms. In their native state, some of their functionalities reside in their material form as hydrogels, thus offering molecular strategies to be exploited. In addition to the various types of interactions mediated by their detailed structure supporting higher-order structure formation, their chemical groups can be readily modified to alter and enhance their functionality, including also the formation of a crosslinked state. Polysaccharides exist either as homopolymers or heteropolymers. In the latter group, variations in the number of constituents, their sequential arrangements and connectivity in terms of branching, can be found and are key in hosting functionality. Polysaccharides also span the range of biocompatible to biological response modifiers, as regulated through their structural detail. There is an increasing interest in carbohydrate-based hydrogel materials, driven by the advantageous properties of these materials.

The goal of this Special Issue is to provide a summary of recent important progress in this field, ranging from basic aspects to applications, thereby highlighting both the diversity and impact of polysaccharides to the hydrogel field. The particularities of the gel state and its formation, and how the molecular detail of the polysaccharides underpins the hydrogel structure formation and final properties, are often challenging to elucidate. The challenge to generate a comprehensive understanding of various facets of the molecular basis for hydrogel structure and formation are best addressed using a multi-tool approach combining information at various length scales. In this Special Issue on “Polysaccharide hydrogels”, researchers from around the world have presented research highlighting various materials, modifications, structures and applications of selected polysaccharide hydrogels. The comprehensive review by Donati and coworkers [1] excellently summarizes the molecular mechanism playing a role in the formation of physical crosslinks of polysaccharide materials using chitosan and its derivatives as the material for reference. Thus, the authors link the nature of the hydrophobic effects, hydrogen bonding capacities and electrostatic-mediated interaction to molecular groups and polysaccharide structure and extrinsic (solution) factors, and extend the electrostatic aspects from the small ion-mediated interactions to higher molecular weight counterparts as encountered in complex coacervation. The contribution by Alves and coworkers [2] adds to these general facets by reporting on the addition of urea to an alkaline aqueous solution to affect the solubility and gelation properties of cellulose.

The two contributions from Yuguchi [3] and Bassett [4] and their coworkers provide insight into the polysaccharide gelation processes at longer length scale. In the first of these two, it is reported that amylose displayed increased crystallinity and gelation following in situ neutralization from an alkaline aqueous solution, whereas structural information related to alginate chains displaying junction zone formation mediated by in situ release of Ca^2+^ was reported on the latter. The publications by Strand [5] and Lapitsky [6] and their coworkers illustrate the relation between polysaccharide modification and mechanical properties and how gelation pathways may impact overall gel stability as assessed at a macroscopic length scale. The alginate modification reported aimed at enhancing the functionality of the alginate in applications such as drug delivery or tissue engineering while preserving the preferable ionic, often Ca^2+^-mediated, gelation properties. The three contributions by Groll [7], Ritz [8] and Kwiecien [9] and their coworkers focus more on the application of the polysaccharide hydrogels. Groll and coworkers [7] describe the development of hyaluronic acid-based bioink for 3D bioprinting by oxidizing the polysaccharide and combine this with a bifunctional crosslinker-forming Schiff base with the modified hyaluronic acid and apply this in the extruding process. Ritz and coworkers report on the application of photo-crosslinked dextran hydrogel as a carrier system of cells that can be used in the context to stimulate bone regeneration. This approach illustrates the need to include biological aspects in the material selection, for which selected polysaccharides often are the preferred choice in many applications. The report by Kwiecien [9] indicates that polysaccharide-based immobilization is also a viable strategy in probiotic delivery strategies where the material integrity over a wider range of conditions are essential for the successful application. As a transition to more sustainable materials exploitation is evident, the contributions to research as in the present Special Issue do indicate that polysaccharides offer a versatile approach that can be expected to aid also in this respect.
